# Programmatic Mapping: Providing Evidence for High Impact HIV Prevention Programs for Female Sex Workers

**DOI:** 10.2196/12636

**Published:** 2019-06-06

**Authors:** Faran Emmanuel, Navindra Persaud, Sharon S Weir, Parinita Bhattacharjee, Shajy Isac

**Affiliations:** 1 Centre for Global Public Health Winnipeg, MB Canada; 2 Family Health International 360 Washington DC, WA United States; 3 University of North Carolina Chapel Hill, NC United States; 4 Partners for Health and Development in Africa Nairobi Kenya

**Keywords:** programmatic mapping, PLACE, size estimation, female sex workers, HIV prevention, microplanning, key populations

## Abstract

Programmatic mapping (PM) is a rapid and efficient mechanism to develop size estimates of key populations including female sex workers (FSWs) and geolocate them at physical locations in a systematic and scientific manner. At the macro level, this information forms the basis for allocating program resources, setting performance targets, and assess coverage. At a micro level, PM data provide specific information on hot spots, estimates of FSWs at those spots, and hot spot typology and days and times of operation, all of which provides targeted service delivery strategies. This information can provide a reliable platform to plan HIV prevention and treatment services to considerable scale and intensity. Above all, the entire PM process requires deep involvement of FSWs, which increases community ownership of the data and can lead to an increased uptake of services. Despite a few limitations, the approach is versatile and can be used in varied country contexts to generate important information about sex work and its dynamics. In this paper, we describe experiences and lessons learned from using evidence generated from PM of FSWs in multiple countries to develop HIV prevention programs at scale.

## Introduction

Female sex workers (FSWs) are disproportionately affected by the HIV epidemic [1]. Globally, 15% of the HIV infections among women may be attributed to sex work [2]. Primary prevention remains the key to epidemic control [3] as available evidence shows that targeted interventions implemented at scale among FSW can halt HIV transmission and reverse the epidemic [4-7]. Programs most likely to reduce new infections are community-owned, grounded in evidence and use a mix of biomedical, behavioral, and structural interventions [8,9].

Acknowledging local epidemic diversity, the Joint United Nations Programme on HIV/AIDS (UNAIDS) Practical Guidelines for Intensifying HIV Prevention recommend the use of local data to inform programs [10,11]. This includes *knowing the epidemic* to develop a clear understanding of the epidemic and tailoring “the response” based on a combination prevention strategy. Moreover, HIV prevention programs tailored to FSWs should include active outreach, engagement, mobilization, and empowerment of FSWs to reduce their vulnerability [12,13]. Thus, for programs to be effective and accountable, they need to prioritize FSWs that can be directly reached through peer outreach and geographically located services [14]. The recently launched HIV prevention 2020 roadmap recommends a “location-population approach” that addresses the heterogeneity of the HIV epidemic and ensures effective and efficient planning and delivery of HIV prevention services [15].

More recently, The Global Fund, the World Bank, World Health Organization (WHO) and UNAIDS, as well as various national programs have successfully used programmatic mapping (PM) approaches to estimate size, geo-distribution, operational typologies, and structural determinants of various key populations (KPs) [16]. Although various size estimation methods provide an absolute number of the key vulnerable population [17,18], PM produces estimates at the level of each hot spot and also assesses the amount of overlap between various spots. Thus, population size estimates derived from PM are adjusted for potential duplication and also provide a consequential distribution of KP members at different spots. In addition to identifying key locations and quantifying risk populations, mapping results can be used to identify existing programs and quickly institute services where they were unavailable. Thus, it provides site-level data in a timely manner to inform program design, monitor the effect of programs, and guide programs to meet the changing needs of FSWs.

The knowledge derived from PM has been used abundantly to provide a reliable platform to plan preventive and treatment services for FSWs. In this paper, we describe experiences and lessons learned from using evidence generated from PM of female sex workers in multiple countries to develop HIV prevention programs for effective coverage at scale.

## What is Programmatic Mapping?

PM [16] is a systematic and scientific approach that identifies locations (often called sites, venues, or hot spots) where KPs engage in risk and provides the local and national population size estimation data necessary to plan prevention and treatment services at appropriate scale and intensity. Programmatic experience in diverse settings show that although a few FSWs operate through hidden networks, a larger proportion congregate and/or meet clients in definable geographic settings and targeting these spots for program delivery is an efficient prevention strategy [19]. To identify where to reach FSWs, PM engages the FSW community as coimplementers to estimate the number of FSWs, describe their geo-distribution and operational typologies, and inform the design and placement of HIV prevention programs to maximize coverage [20,21]. PM includes profiling locations where FSWs can be reached and assesses service availability in those locations or nearby. The term “programmatic” underscores PM’s focus on generating information useful to program design and improvement.

### How Programmatic Mapping Works

During PM fieldwork, the study area is segmented into zones, and an exhaustive list of locations where FSWs congregate is systematically compiled within each zone. Subsequently, these spots are visited with the assistance of local community members (FSWs) to confirm onsite risk activities and the number of FSWs at each location. Population size is estimated at the site level, which is then adjusted to account for double counting and/or mobility across sites, to finally reach an estimated number of FSWs in the study area. Planning for and calculating coverage requires an understanding of where sex work happens and an estimate of various subtypes in each of these locations and their characteristics. One of the key strengths of this approach lies not only in its development of size estimates but also in providing a distribution of FSWs at different hot spots. For planning services and subsequently monitoring coverage, this approach has been proven valuable in ensuring high level coverage [22].

PM includes 3 main approaches: basic geographic mapping (GM), the Priorities for Local AIDS Control Efforts (PLACE) method, and progressive mapping. Selecting the best approach depends on the context and the availability of time and resources. However, all adhere to the principles of generating location and population size information to design and implement programs, and community ownership and leadership. Although basic GM has been used primarily for mapping and estimating size of KPs, PLACE has been used pragmatically to identify locations where persons meet new partners, and thus, networks where HIV transmission is most likely to occur. Both GM and PLACE have been used where mapping and size estimates are not available and/or more rigorous baseline or follow-up estimates of program coverage are required. Progressive mapping approach is implemented as a component of a more mature program, where it is used quickly by program teams to generate information to scale up programs. A practical difference between the methods is that GM and PLACE include systematic interviews with key informants across all study zones to develop an exhaustive list of sites, whereas progressive mapping uses a crude list of sites from previous mapping as the starting point, available program data, and discussions with peer educators and outreach workers. Each method includes visits to identified spots to verify they are operational, describe their characteristics, and collect information on estimates, typology, and other operational characteristics of the hot spot. This is done by interviewing a key informant, preferably an FSW who operates from the identified spot, or someone knowledgeable about the spot (eg, a bar manager). Both GM and PLACE have extended the value of basic PM by using the venue list as a sampling frame for venue-based biobehavioral surveillance. Examples have been provided in subsequent sections on how countries with large FSW populations such as India, Kenya, and Haiti have used these approaches to gather sufficient information in a timely manner with enough geographic specificity and at low cost to plan KP program scale-up [23-25]. Other critical information such as condom availability or service availability for FSWs in the specific spots can be also collected in all the methods if such information is needed for effective program planning.

**Figure 1:**
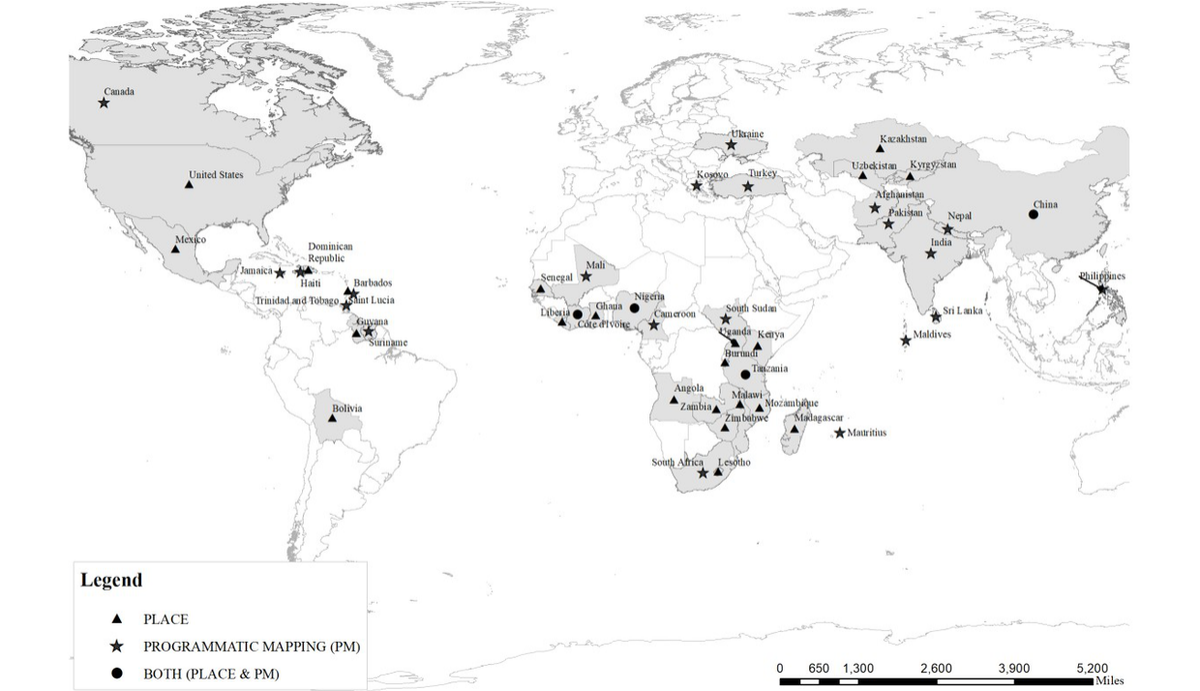
Countries where various forms of programmatic mapping have been implemented.

Development partners (eg, The Global Fund, the World Bank, the Bill and Melinda Gates Foundation, WHO/UNAIDS, and PEPFAR) have supported various PM approaches in a number of countries. Figure 1 shows countries where various forms of PM have been implemented.

## Using Programmatic Mapping for Development of Prevention Programs

There are a number of ways PM data can be used to develop strategic plans for targeted HIV prevention among KPs including FSWs and rapidly establish appropriate programs and basic services. In addition, PM has been used extensively to estimate the size of FSW populations, identify prevention coverage gaps, and quickly institute services where they are nonexistent. The following section presents some examples of how PM data have been used in various countries to strategize, develop, and implement FSW programs.

### Use of Programmatic Mapping Data at a Macro Level to Develop National and Subnational Program Plans

Conceptually, high impact programs aim to reach the highest number of FSWs with quality services. At the macro level, national and subnational programming [23] focuses on establishing HIV prevention and care services, upscaling programs where needed, and launching new programs where they are nonexistent. Effective program coverage can only be measured when a realistic estimate of the number of FSWs is available.

PM data in Kenya estimated 103,298 FSWs in 7 provinces [26], which was used by National AIDS and STIs Control Program as a denominator to set national coverage targets for the minimum package of services defined in the national guidelines [27]. PM results guided decisions about where to prioritize services to improve program coverage. Thus, Nairobi (with 27% of the FSW population) was the top priority for prevention efforts, followed by Coast, Rift valley, Nyanza, and Western province. Within Nairobi, the subcounty of Starehe, with 25% of the Nairobi FSW population, was prioritized over counties with lesser concentrations of FSWs [26]. PM data also inform progressive coverage based on resources and targets and provide insights into the heterogeneity of site typology and overall sex work so that intervention packages can be tailored to the local context [25,28]. For example, PM in Nigeria identified diverse sex work typologies based on the operational dynamics of sites and sex workers [29]. Thus, as 80% of the FSWs were venue-based in Abuja, the program focused on robust outreach and service delivery efforts at bars and night clubs and implemented less intensive programming with FSWs at other type of spots. On the basis of the number of FSWs (Lagos: 46,691 vs Anambra: 4846), 10-fold more resources were allocated for the FSW prevention program in Lagos. The geographical distribution of FSWs in Mauritius highlighted that expanding HIV prevention services in only 04 of the 09 districts would provide coverage to more than 80% of the FSW population in Mauritius, targeting specifically the high-intensity sites, thus, improving outreach and coverage within available resources [30].

### Use of Programmatic Mapping Data at a Micro Level to Enhance Outreach and Services

Micro planning decentralizes outreach management and planning to a grass-root level of outreach workers (ORW) and peer educators (PEs) through a process that collects and uses data at an individual level to empower a community to make healthy decisions for themselves [31]. Although macro plans are made at the national or subnational levels, micro level planning takes place at the site level and becomes the cornerstone of peer-led programs [32].

Through PM, a list of all locations or hot spots where FSWs operate is developed, assigning a range of estimates for FSWs at each spot by subtypology (eg, street, venue, and public place). Furthermore, PM data characterize the spots by providing information on timing of sex work activity at the spot (busiest time of the day or busiest day of the week), which is used as the basis for micro planning. Steps in micro planning include “hot spot validation” identified by PM, followed by “spot load mapping” where FSWs’ peers validate and record peak times and days of operation along with other characteristics of the spot. This is done to build ownership of the PM data by the peers, update spot lists, and estimate the number of PEs required. “Contact mapping” is then done to understand FSW networks and select PEs and determine their allocation across the target area, taking spot clustering in account. “Peer plans” are developed by PEs for each allocated FSW based on her risk and vulnerability, prioritizing those with higher risk and vulnerability. Finally, “Peer calendars” are used to document services provided to each FSW based on individual peer plans and track them at each visit outreach and micro planning [31,32]. The entire process of micro planning was conducted in Haiti using the PM data generated using the PLACE approach. These tools assisted the program to recognize each sex worker as an individual, assess her HIV risk (client volume, age, duration in sex work) and vulnerability (violence), and help plan a prevention pathway for her [33].

Likewise, in Mombasa, Kenya, PM data supported successful scale-up of HIV programs for FSWs. PM identified 9208 FSWs with the highest concentration (5809) in Kisauni and Mvita [26]. Hot spot validation and spot load mapping informed that 60% of FSWs operated through venues such as bars and clubs and another 30% through street spots. The 233 validated spots were plotted on a map, highlighting the number of FSW congregating at each spot. Location, spot size, and typology were used to allocate spots to PEs within their area of operation. On the basis of a ratio of 1 PE for 60 to 75 FSWs, 68 PEs were recruited, and 12 ORWs were hired to supervise them. Within areas of high FSW concentrations, 2 drops in centers and clinics were established, and linkage with government and NGO clinics were established for FSWs in dispersed or far away spots. Through peer plans and peer calendars, cumulative enrollment increased to 80% of the estimated FSW population within the first 10 months. An average of 35 to 40 condoms per month were distributed to each FSW during outreach as per the client volume calculated at registration [24]. As shown in the example, sex worker programs benefit tremendously from PM data in its finest details to estimate the specific number of condoms, lubes, outreach testing supplies, and other materials needed. Not only does this improve the efficiency and effectiveness of programs, it has proven its value in strengthening community networks through a community-based approach, which adds to the ownership of such programs [34].

### Use of Programmatic Mapping Data to Estimate Population Size

PM has been used extensively in a number of countries to produce size estimates of sex worker populations [25,26,29,30]. Mapping produces estimates of FSWs at each spot, assesses the amount of overlap between various spots, and then adjusts the total number for potential duplication to calculate a range of estimated number of FSWs in a focused geography. Although PM provides valid population size estimates in a timely manner to inform program design and supports monitoring and evaluation of ongoing interventions, a few limitations of the approach need to be discussed. As the inherent approach focusses on geolocation-based sex work, there is a probability to miss out FSWs who do not visit physical venues and solicit online or via social media. Thus, care needs to be taken while relying on size estimates derived from PM approaches alone. Data from PM are best used as a starting point for programs, and it is anticipated that as programs implement, they will validate and supplement the information and thereby periodically update the estimates [23,24,33]. Despite this limitation, PM has been used extensively across countries for estimating size of the KPs successfully as part of a National size estimation exercise [25,26,30].

### Use of Programmatic Mapping Data for Monitoring and Research

Finally, PM data provides service delivery programs with denominators, which are crucial to be able to set goals and establish benchmarks for key outcomes indicators. Key indicators related to program coverage and utilization of programs and services by target populations serve as markers for program success. Coverage gaps at spot level can be evaluated through mapping of these spots on a continuous basis. PM data provide denominators for monitoring 90-90-90 targets and service uptake indicators across the HIV prevention, care, and treatment cascade among FSWs. In Kenya, the national program routinely uses PM data as a denominator to measure the KP program performance every quarter [35]. Under the LINKAGES project, the list of spots generated through PM contributed to the development of a sampling frame for conducting integrated bio-behavioral surveys (IBBS) of KPs in Haiti, Malawi, Angola, and Mozambique, which helped development of 90-90-90 indicators, including proportion of FSW on treatment with suppressed viral loads [25,36-38].

Likewise, Pakistan has extensively used PM data to sample KPs for IBBS. In each city, the IBBS sample was distributed based on weights derived from the respective number of KPs in each zone. Spots were randomly selected from spot lists generated by PM within each zone, and later, respondents were selected based on sampling weights assigned to each spot based on spot size, making the sample representative of the overall population [39,40]. A similar approach was employed across India, where PM data were used to select a random sample of hot spots and select respondents from the selected hot spots. As PM listed all hot spots in a selected geography, the list of spots served as a sampling frame, and it was possible to select a representative sample of KPs using a Time Location Cluster Sampling technique [41]. Thus, PM provides a sampling frame from which a representative sample of FSWs can be selected for survey. Table 1 provides the information available through mapping and its use for service delivery planning.

**Table 1:** Information available through programmatic mapping and its use for service delivery planning.

Information	How this information is used
National size estimates	Develop national strategic plans for HIV prevention, care, and support for key populations
	Decide on national budgetary and resource allocations and costing exercises
	Set denominators for coverage targets for HIV prevention and treatment programs
	Evaluate prevention response at a national level and identify coverage gaps
Subnational size estimates	Help the country to decide where to prioritize programs to improve meaningful coverage
	Decide on resource allocations at a regional or subnational level
	Compare prevention response among regions and identify prevention gaps
	Draw representative samples for key population research by assigning sampling weights based on national distribution of study population
Geo-distribution of spots	Target locations/spots for localizing interventions
	Develop local maps and set a plan for coverage
	Prepare spot clusters and allocate spots to peer educators based on geo-proximity of spots
	Establish locations for health clinics, drop-in centers, HIV testing centers etc, as well as condom and lubricant distribution channels
Number and size of spots	Target locations/spots for localizing interventions
	Decide which spots to prioritize and focus to match coverage targets
Operational dynamics of a spot; peak days and peak times of operations	Determine human resource needs, that is, how many peer educators and outreach workers are needed to adequately cover the population. For example, 1 peer educator could work with 50-60 FSWs^a^, whereas 1 outreach worker can manage working with 5-6 peer educators.
	Size estimates provided at the spot level can be used to estimate the specific number of condoms, lube, outreach testing supplies, and other materials needed
	The human resource and commodity plan needs to be based on peak estimates so that no one remains uncovered
	Peak times and peak days might be utilized to determine time of outreach
Operational typology of spots/FSWs	Inform intervention design based on the subtypology of spots and FSWs (eg, brothel, street, bar, night club, massage parlor based, and home-based FSWs).
Use in research	Mapping data is used as a sampling frame for national level surveys, including integrated bio-behavioral surveys.

^a^FSWs: female sex workers.

## Conclusions

PM is a low-cost and reliable approach designed to generate the most critical information required for planning and implementing effective HIV prevention, care, and treatment programs. PM data have been used at national, provincial, district, subdistrict, and spot levels. At a national level, PM informs funding requirements and resource allocation, program scale-up, target setting, and coverage assessment. At a subnational level, PM prioritizes geographies where interventions should be scaled up to ensure the highest coverage possible within the available resources. Saturating coverage of KPs in high concentration geographies is preferable to spreading services thinly across a wider area. At the district or town level, PM assists programs to assess resources required for outreach and clinical staffs and for commodity requirements such as number of condoms/lube and HIV testing kits. At a spot level, PM enhances outreach to ensure the right number of peer ORWs are available to saturate coverage as well as allocate spots to the most ideal PE. PM further improves program design by carefully evaluating typologies and functional timings of the spots and establishes denominator to monitor access to services and coverage at each spot.

Despite a few limitations, PM provides essential information about the size, distribution, and characteristics of FSWs in a systematic and scientific manner. Above all, the process relies substantially on the strength and involvement of civil society and community organizations that represent and are engaged with KPs. This practically increases the appropriateness and ownership of program design and implementation and results in increased uptake and more efficient and effective programs based on the needs and priorities of the FSW community.

## Abbreviations

FSWs: female sex workers
GM: geographic mapping
IBBS: integrated bio-behavioral survey
KPs: key populations
ORW: outreach worker
PE: peer educator
PLACE: Priorities for Local AIDS Control Efforts
PM: programmatic mapping
UNAIDS: Joint United Nations Programme on HIV/AIDS
WHO: World Health Organization
